# Supplementary material for: Preclinical validation of a live attenuated dermotropic Leishmania vaccine against vector transmitted fatal visceral leishmaniasis
Source: Commun Biol. 2021 Jul 30;4:929. doi: 10.1038/s42003-021-02446-x (PMC8324786; doi:10.1038/s42003-021-02446-x)
Supplement: Supplementary file 2 — Supplementary Information [file 42003_2021_2446_MOESM2_ESM.pdf]

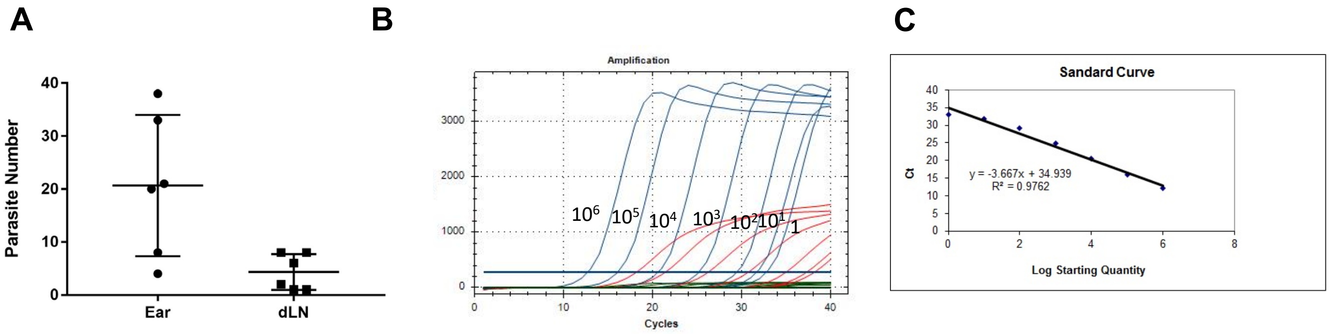

Supplementary Figure 1: Determination of parasite load by RT-PCR at 49-days of post-injection :

(**A**-**C**) Ear and dLN parasite burden of *LmCen*$^{-/-}$ immunized hamsters (n=6) at 49 days of post inoculation was determined by RT-PCR (A). Amplification of *L. major* parasites from DNA co-extracted after spiking hamster ears with known numbers of parasites (B). The parasite burden in the ear and dLN samples from *LmCen*$^{-/-}$ *group* was quantified by generating a standard curve of the cycle number (Ct) that corresponds to the number of spiked *L. major* parasites (C).

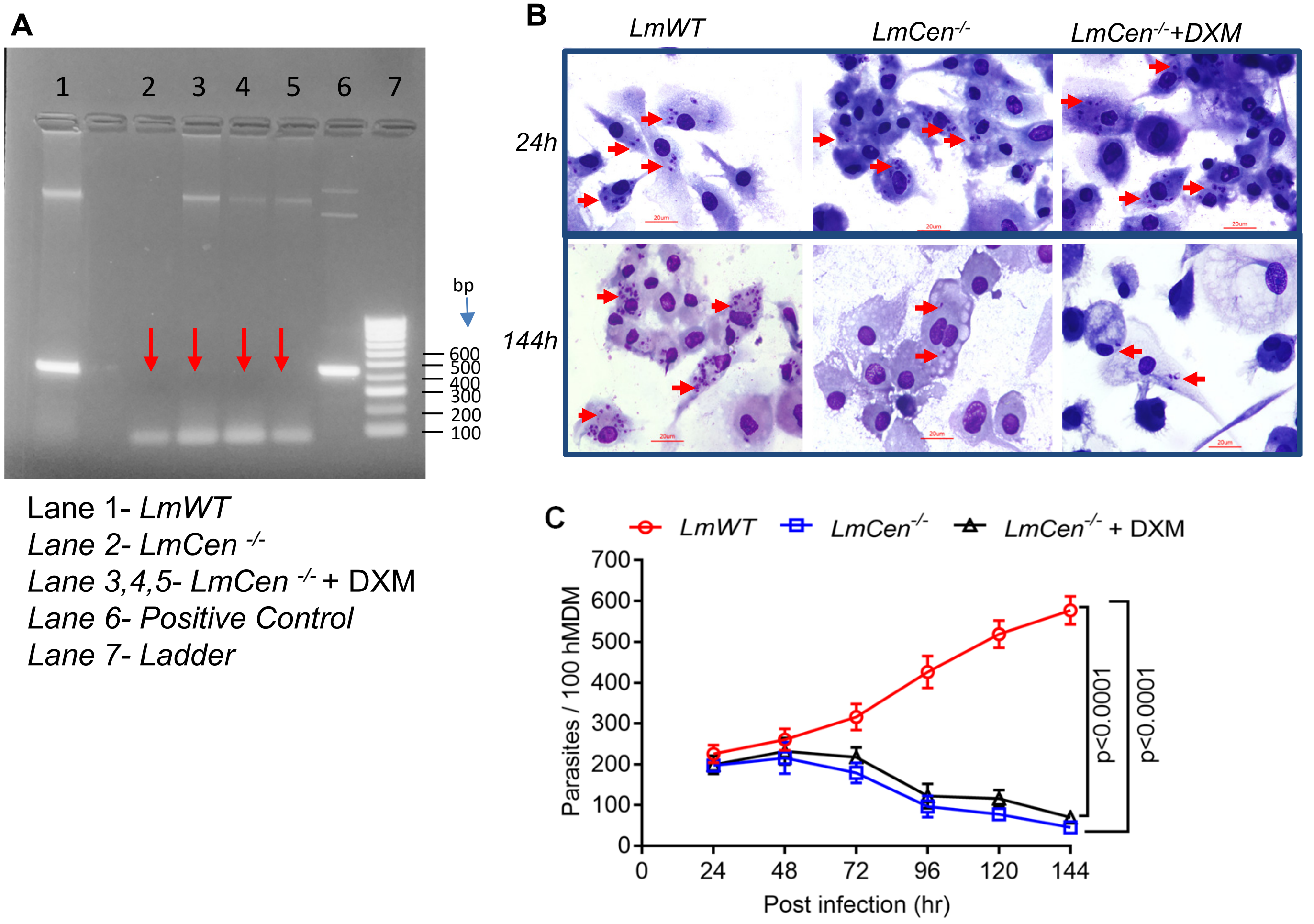

Supplementary Figure 2. ***LmCen*$^{-/-}$ does not cause any lesions even in immune suppressed animals** (**A**) 1% Agarose gel electrophoresis results for the characterization of *LmCen*$^{-/-}$ parasites isolated from *LmCen*$^{-/-}$ plus DXM treated group using *L. major centrin* gene specific primers. Lane-1, PCR results from the genomic DNA of parasites isolated from *LmWT*, Lane-2, PCR results from the genomic DNA of 2 parasites isolated from *LmCen*$^{-/-}$ treated group, Lane-3,4,5 PCR results from the genomic DNA of parasites isolated from *LmCen*$^{-/-}$ +DXM treated group, Lane-6 PCR results from the plasmid DNA containing *centrin* gene as a positive control . Lane 4 , 100 bp DNA ladder (Bioline). Red arrow indicates the absence of main product bands (*centrin* gene) of 450bp in Lane-2,3,4 and 5. (**B** and **C**) Photographs (B) of human monocyte derived macrophages (hMDM) at 24h and 144h post-infection with parasites isolated from *LmCen*$^{-/-}$ plus DXM treated group as well as *LmWT* and *LmCen*$^{-/-}$ parasites respectively. The number of amastigotes (red arrow) was determined microscopically up to 144h post infection. The data (Mean ± SD) are represented as the number of parasites per 100 hMDM (C) of one independent experiment (p values were determined by Unpaired two-tailed t test).

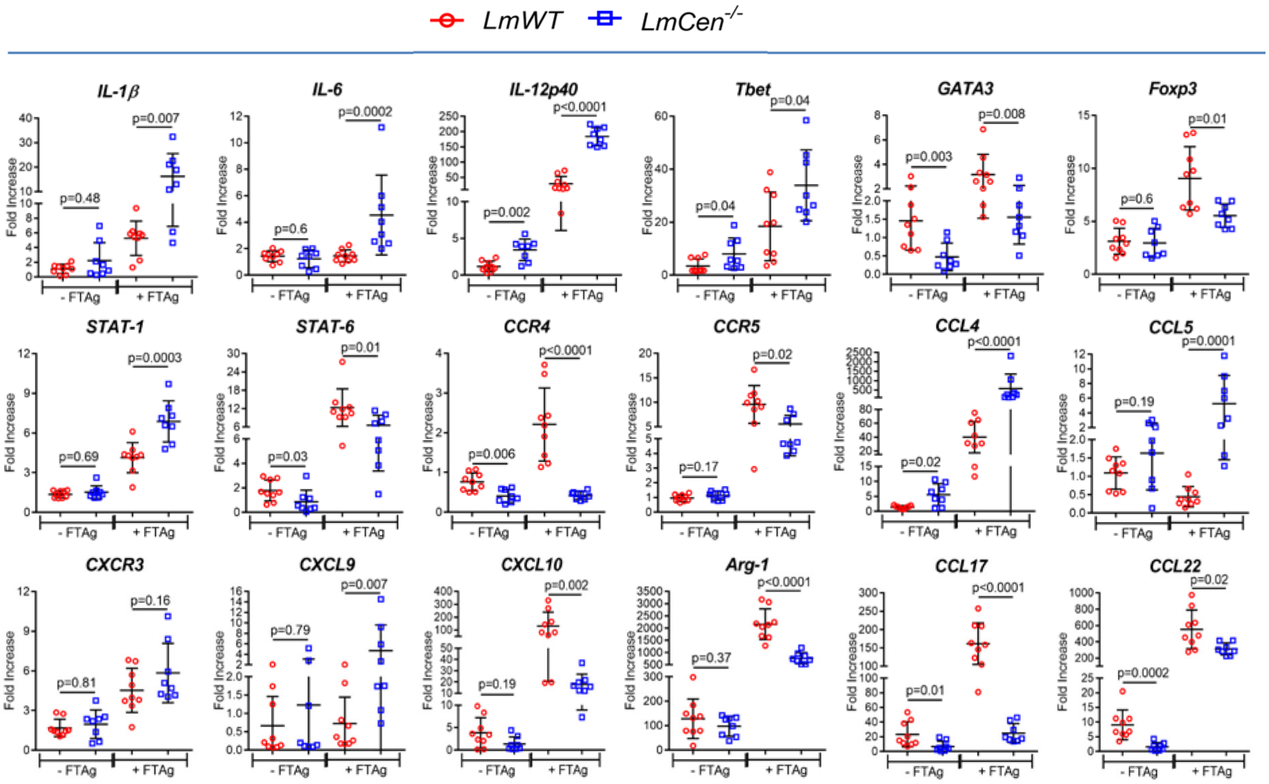

Supplementary Figure 3. *LmCen*$^{-/-}$ immunization induces pro-inflammatory immune response. The expression of transcripts (IL-1β, IL-6, IL-12p40, T-bet, GATA3, Foxp3, STAT-1, STAT-6, CCR4, CCR5, CCL4, CCL5, CXCR3, CXCL9, CXCL10, Arg-1, CCL17 and CCL22) was confirmed by RT-PCR in the spleen (with or with-out 24h of *L. major* freeze thaw antigen re-stimulation; ± FTAg) of hamsters immunized with *LmCen*$^{-/-}$ parasites (n=8) and compared with *LmWT* (n=9) infected group following 7 weeks of post inoculation. The data were normalized to either γ-Actin or 18S expression. Results (mean ± SD) are representative of cumulative effect of two independent experiments. Statistical analysis was performed by non-parametric Mann-Whitney two-tailed test.

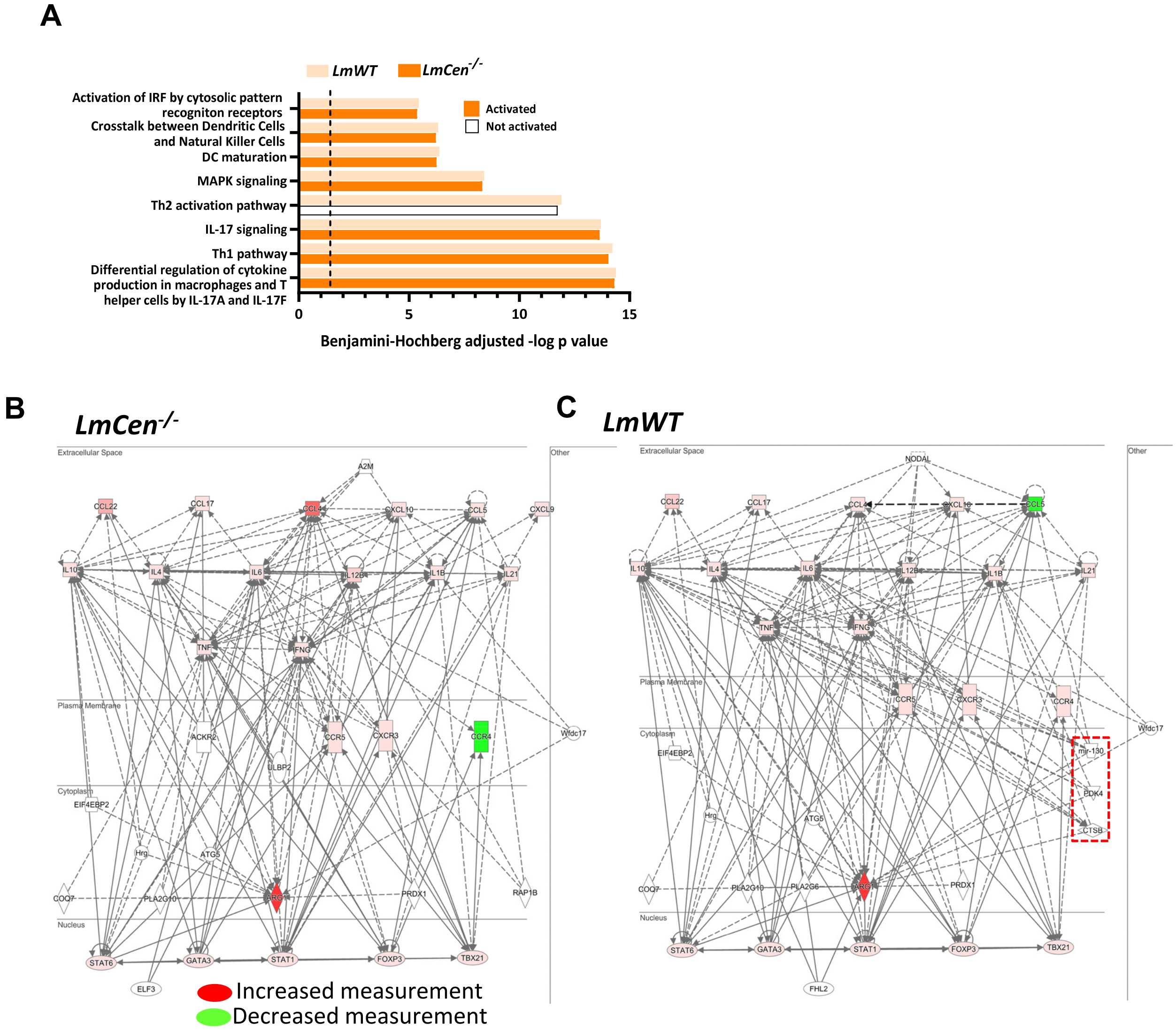

Supplementary Figure 4: Immunogenicity of *LmCen*$^{-/-}$ parasites in hamsters

A, Ingenuity pathway analysis comparing the expression of various Th1/Th2 associated immune markers showing common canonical pathways activated in *LmWT* or *LmCen*$^{-/-}$ infections. **B** and **C.** Immune signaling networks observed in *LmCen*$^{-/-}$ (B) and *LmWT* (C) infections show differential expression of immune markers in two infections. The pink, green and red color indicates experimentally measured markers and colorless nodes represent IPA predictions. Datasets from RT-PCR experiments represented in Supplementary Figure 3 were used in the IPA analysis.

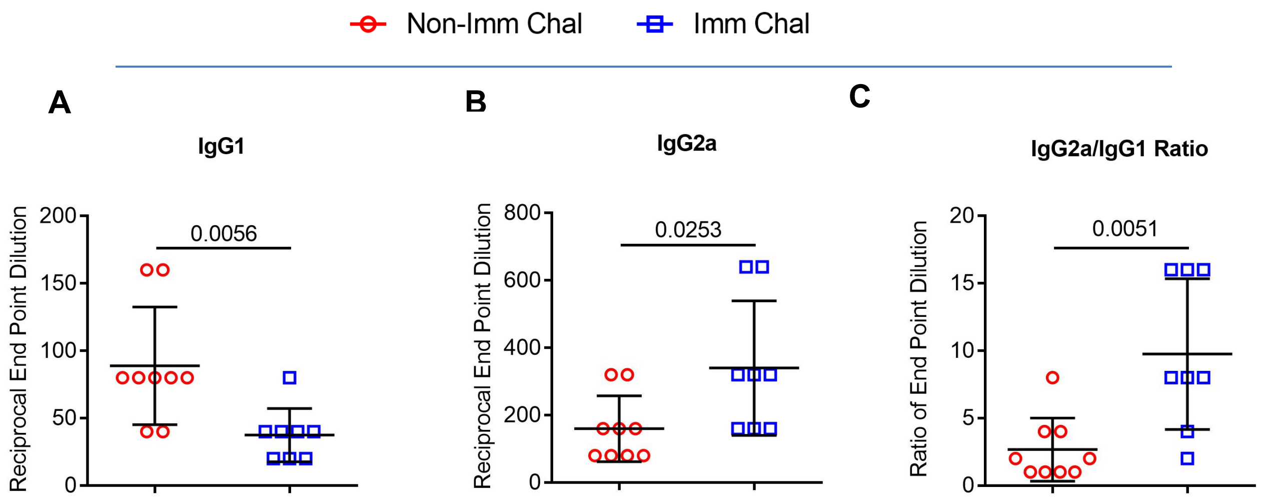

Supplementary Figure 5. ***LmCen$^{-/-}$*** **immunization induces IgG2a type of immune response.**
A, B, Anti-leishmanial IgG1 (A) and IgG2a (B) were measured from the serum of hamsters immunized with *LmCen$^{-/-}$* parasites (n=8) and compared with *LmWT* (n=9) infected group following 7 weeks of post inoculation. (C) The ratio of IgG2a/IgG1. Results (mean ± SD) are representative of cumulative effect of two independent experiments. Statistical analysis was performed by non-parametric Mann-Whitney two-tailed test.

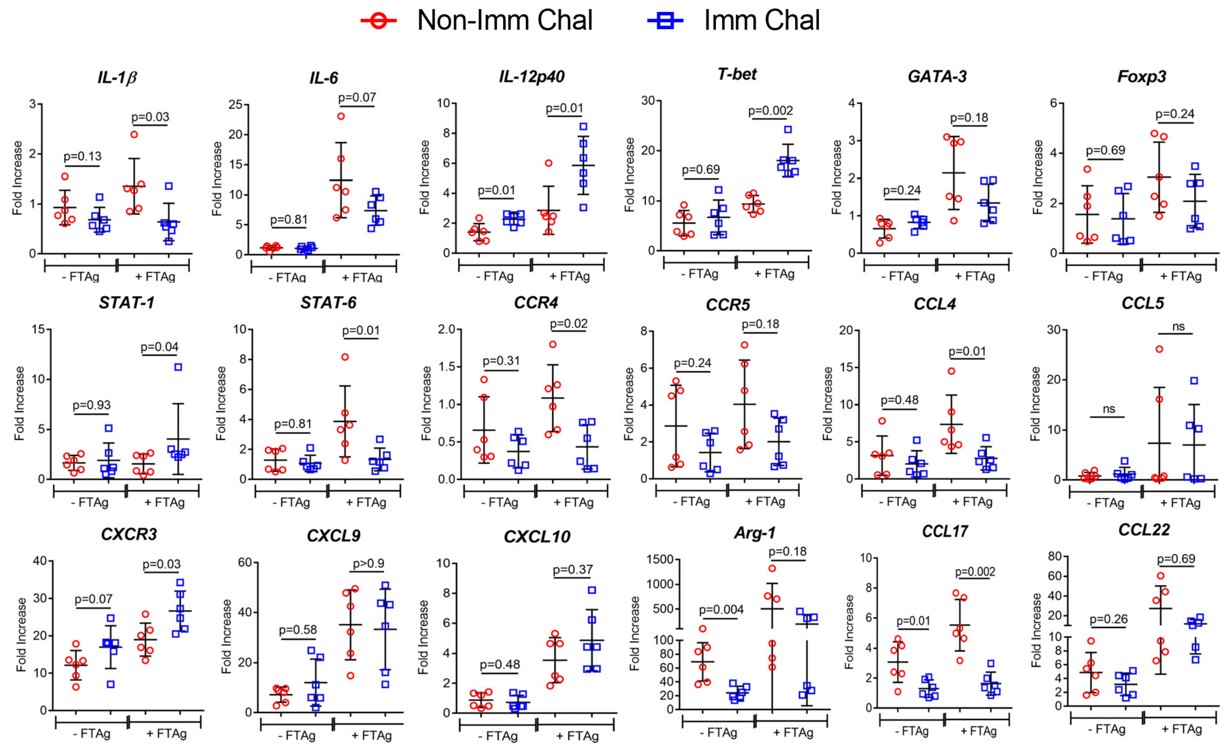

Supplementary Figure 6. *LmCen*$^{-/-}$ immunized hamster induces pro-inflammatory type of immune response upon challenge with wild type *L. donovani.*

The expression of transcripts (IL-1β, IL-6, IL-12p40, T-bet, GATA3, Foxp3, STAT-1, STAT-6, CCR4, CCR5, CCL4, CCL5, CXCR3, CXCL9, CXCL10, Arg-1, CCL17 and CCL22) was confirmed by RT-PCR in the spleen of (with or with-out 24h of *L. donovani* freeze thaw antigen re-stimulation; ± FTAg) of *LmCen*$^{-/-}$-immunized (Imm Chal, n=6) and age matched non-immunized (Non-Imm Chal, n=6) hamsters at 1.5 months of post *L. donovani* needle challenge. The data were normalized to either γ-Actin or 18S expression. Results (mean ± SD) are representative of cumulative effect of two independent experiments. Statistical analysis was performed by non-parametric Mann-Whitney two-tailed test.

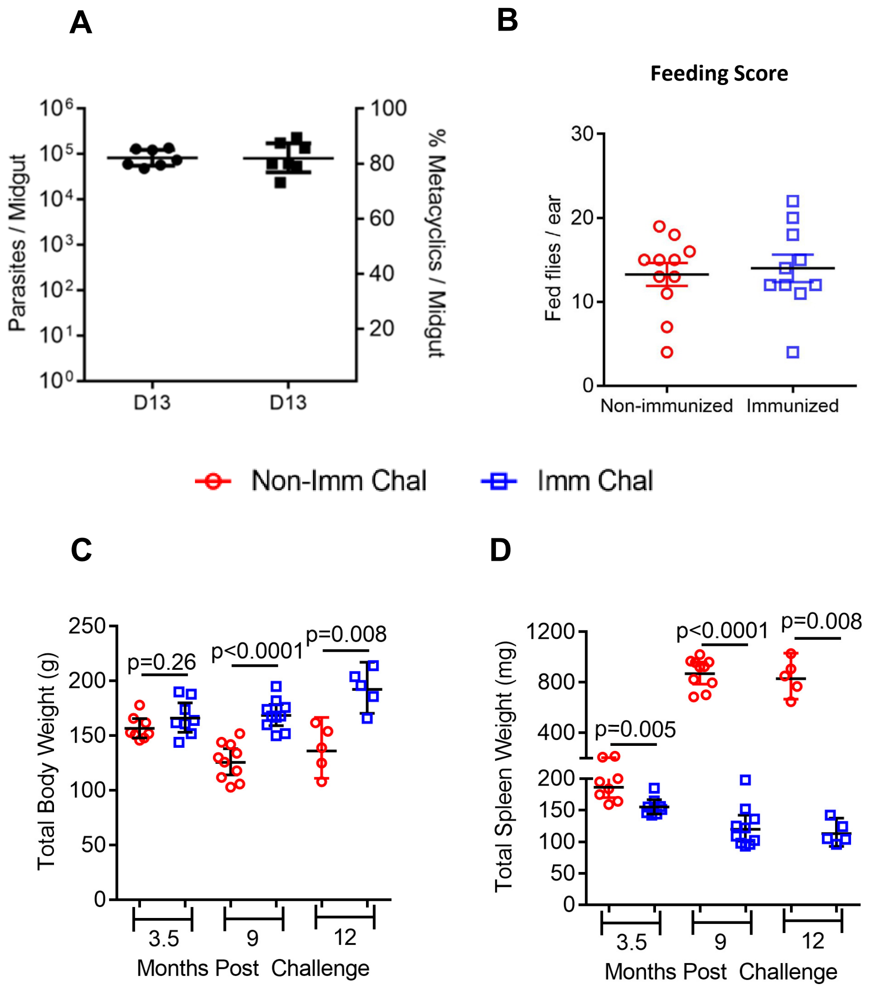

Supplementary Figure 7: Parasite burden, percent of metacyclic, feeding score of sand flies used for *L. donovani* infection and body and spleen weight of hamsters.

(**A**) Assessment of the parasite load and percent metacyclics per sand fly midgut after 13 days of post sand fly infections. For challenge studies, 13 days of post infected sand flies were used. (**B**) Post transmission feeding score from 30 total sand flies exposed to right ears of each hamster. Data from one of the two independent experiments is shown. (**C** and **D**) Body weight (gms) (**C**) and spleen weight (mg) (**D**) of *LmCen*$^{-/-}$ immunized (Imm Chal) and age matched non-immunized hamsters (Non-Imm chal) were determined after various periods (3.5-, 9- & 12- months) of *L. donovani*-infected sand flies (*Lutzomyia longipalpis*) challenge. Results are representative of cumulative effect of two independent experiments for 3.5 and 9 months post-challenge and one experiment for 12 months post-challenge. Bars represent the geometric means with 95% Cl of total 5-10 hamsters (n=8, n=10 and n=5 for 3.5M, 9M and 12M post challenge respectively of non-immunized and immunized challenged groups) in each group are shown. Statistical analysis was performed by non-parametric Mann-Whitney two-tailed test.

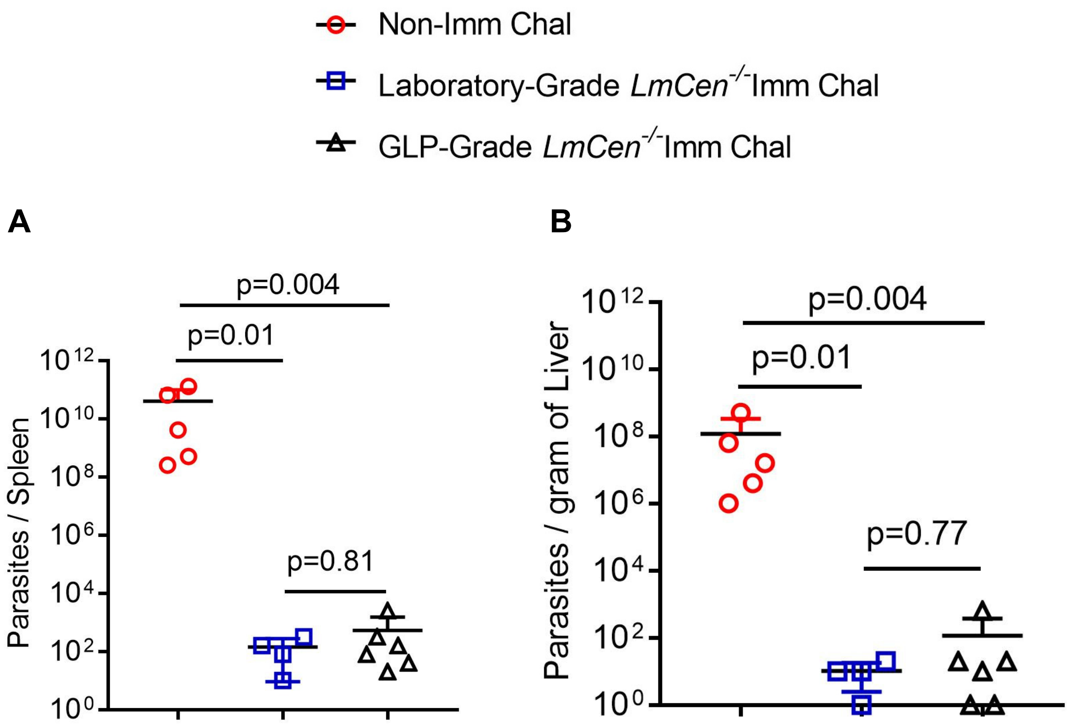

Supplementary Figure 8: Protective efficacy against *L. donovani* following 9-month post needle challenge.

(**A** and **B**) Parasite loads in the spleen (A) and liver (B) of hamsters either immunized with laboratory-grade (Laboratory-Grade Imm Chal, n=4) or GLP-grade (GLP-Grade Imm Chal, n=6) *LmCen*$^{-/-}$ parasites or age matched nonimmunized control (Non-Imm Chal, n=5) were determined by limiting dilution following 9 months of post needle challenge with *L. donovani* and expressed as number of parasites per Spleen and per gram of Liver. Results (Mean ± SD) are representative of one experiment (p values were determined by Mann Whitney two-tailed test).

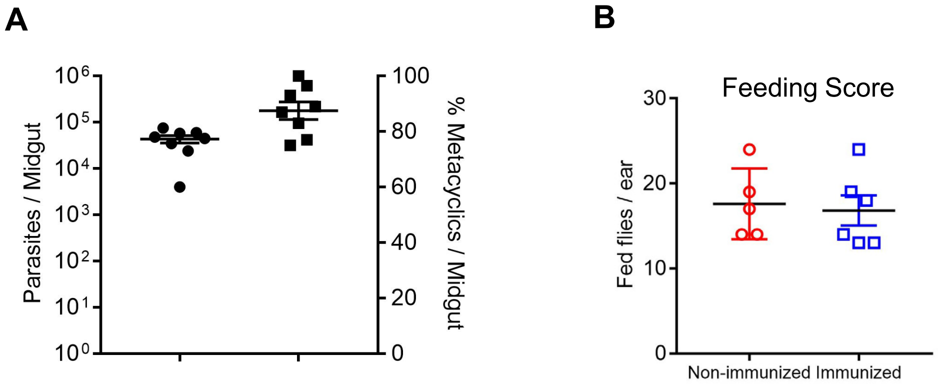

Supplementary Figure 9: Parasite burden, percent of metacyclic, feeding score of sand flies used for *L. donovani* infection transmitted by sand fly bites.

(**A**) Assessment of the parasite load and percent metacyclic per sand fly after 13 days of post sand fly infections. For challenge studies, 13 days of post infected sand flies were used. (**B**) Post transmission feeding score from 30 total sand flies exposed to right ears of each hamster.

Supplementary Table 1: Fold change ratio of normalized expression of the genes in spleens of LmCen$^{-/-}$ infected hamsters relative to naïve hamsters

LmCen$^{-/-}$ versus Naïve

ID	FC ratio	p.value	
IL4	2.96625	0.000452	
IL10	6.8075	0.000504	
IL21	7.44125	4.23E-05	
IL12B	183.9775	9.16E-12	
IFNG	45.44	8.78E-07	
TNFA	34.12375	0.001605	
IL1B	16.21875	0.000188	
IL6	4.53875	0.003006	
TBX21	33.91125	2.22E-06	FC ratio is the average of the LmCen$^{-/-}$ samples divided by 1
GATA3	1.555	0.037445	p.value is calculated by the T.TEST() function with an example below
Foxp3	5.53782	3.04E-09	=T.TEST(LmCen$^{-/-}$ samples range, Naïve samples range, 2, 2)
Arg1	771.9813	3.72E-08	
CCR4	0.42125	3.23E-11	Once uploaded to IPA, use the 'infer observations' button
CCR5	5.5875	1.06E-06	Set FC ratio to 'expr ratio' and p.value to 'expr p-value'
CCL4	573.0738	0.041134	
CCL5	5.2775	0.004234	
CCL17	24.9695	5.35E-05	
CCL22	311.1659	1.72E-09	
CXCL9	4.7	0.038178	
CXCL10	17.85875	4.5E-05	
CXCR3	5.84375	1.01E-05	
STAT1	6.87875	8.8E-09	
STAT6	6.605	0.000106	

Fold change ratio of normalized expression of the genes in spleens of LmWT infected hamsters relative to naïve hamsters

LmWT versus naive

ID	Expr ratio	p.value
IL4	37.42333	0.000151
IL10	57.67556	3.92E-06
IL21	93.33556	1E-10
IL12B	29.78667	0.003774

IFNG	17.42333	1.7E-05
TNFA	11.23667	0.000857
IL1B	5.277778	0.000116
IL6	1.453333	0.013085
TBX21	18.43222	0.001836
GATA3	3.176667	0.002073
Foxp3	9.062259	1.66E-06
Arg1	2158.238	7.84E-08
CCR4	2.211111	0.002066
CCR5	9.59	1.54E-05
CCL4	39.92444	0.000201
CCL5	0.444444	4.38E-05
CCL17	161.592	8.31E-07
CCL22	551.9522	9.57E-06
CXCL9	0.721111	0.294571
CXCL10	129.6678	0.004595
CXCR3	4.531111	2.57E-05
STAT1	4.134444	1.25E-06
STAT6	12.34111	0.000101

Supplementary Table 2: Upstream regulators predicted by IPA to be activated in LmWT or LmCen$^{-/-}$ infected hamster spleens

LmWT

Upstream Regulator	Expr Fold Change	Predicted A	Activation z-s	p-value of ove	Target Molecules in Dataset
CARD9		Activated	2.204	8.63E-13	IL10,IL12B,IL1B,IL6,TNF
GPR84		Activated	2	2.08E-10	IL12B,IL1B,IL6,TNF
CLEC6A		Activated	2.216	3.44E-15	IL10,IL12B,IL1B,IL6,TNF
DOCK8		Activated	2.449	3.24E-10	CCL4,CXCL10,IL12B,IL6,STAT1,TNF
IL33		Activated	2.776	1.4E-16	ARG1,CCL17,IFNG,IL10,IL1B,IL4,IL6,TNF
CLEC7A		Activated	2.413	6.4E-15	CCL4,IL10,IL12B,IL1B,IL6,TNF
TLR7		Activated	2.865	3.09E-16	CCL4,CXCL10,IFNG,IL10,IL12B,IL1B,IL6,STAT1,TNF
REL		Activated	2.209	1.94E-13	CCL4,CXCL10,FOXP3,IFNG,IL12B,IL1B,IL21,IL4,TNF
ITGA5		Activated	2	4.46E-11	IL12B,IL1B,IL6,TNF
STAT4		Activated	2.557	4.3E-13	CCR5,IFNG,IL10,IL6,STAT1,TBX21,TNF
TLR4		Activated	2.839	4.4E-16	CCL4,CCL5,CXCL10,IFNG,IL10,IL12B,IL1B,IL4,IL6,STAT1,TNF
ITGB1		Activated	2	6.22E-10	IL12B,IL1B,IL6,TNF
TLR2		Activated	2.491	4.18E-25	ARG1,CCL4,CCL5,CCR5,FOXP3,GATA3,IFNG,IL10,IL12B,IL1B,IL4,IL6,TNF
IRAK4		Activated	2.024	4.43E-16	IFNG,IL10,IL12B,IL1B,IL4,IL6,TNF
STAT6	12.341	Activated	2.204	1.38E-16	ARG1,CCL17,CCR5,GATA3,IFNG,IL10,IL12B,IL4,IL6,STAT6,TBX21,TNF
SAMSN1		Activated	2.449	1.23E-09	CCL4,CXCL10,IL12B,IL6,STAT1,TNF
CDKN2A		Activated	2.044	9.84E-18	CCL22,CCL4,CCL5,CCR4,CXCL10,IFNG,IL1B,IL6,TNF
ICOSLG/LOC1027239		Activated	2.194	8.63E-13	FOXP3,IFNG,IL10,IL4,IL6
CSF2		Activated	2.567	1.44E-14	ARG1,CCL17,CXCL10,IL10,IL12B,IL1B,IL4,IL6,TNF
CD40LG		Activated	2.703	3.46E-16	CCL4,CXCL10,IFNG,IL10,IL12B,IL1B,IL6,TNF
IL2		Activated	2.108	5.25E-19	CCL4,CCL5,CCR5,CXCR3,FOXP3,GATA3,IFNG,IL10,IL12B,IL4,IL6,TBX21,TNF
NOD2		Activated	2.202	1.4E-11	CXCL10,IL10,IL12B,IL1B,IL6,TNF
MYD88		Activated	2.873	8.69E-22	ARG1,CCL17,CCL4,CCL5,CXCL10,IFNG,IL10,IL12B,IL1B,IL4,IL6,TBX21,TNF
SYK		Activated	2.401	5.53E-10	CCL4,CXCL10,GATA3,IL1B,IL21,TNF
PRKCQ		Activated	2.603	1.17E-18	FOXP3,GATA3,IFNG,IL10,IL4,IL6,TBX21,TNF
SELPLG		Activated	2	1.19E-07	CCL4,IL10,IL1B,STAT6
TICAM1		Activated	2.381	1.02E-14	CCL4,CCL5,CXCL10,IL10,IL12B,IL1B,IL4,IL6,TNF
MAPKAPK2		Activated	2.156	1.26E-09	ARG1,CXCL10,IL10,IL6,TNF
FCGR2A		Activated	2.605	2.5E-15	CCL22,CXCL10,IFNG,IL10,IL1B,IL6,TNF
C3		Activated	2	3.73E-10	CCL4,IFNG,IL10,TNF
GATA3	3.177	Activated	2.205	8.27E-18	CCL4,CCR5,FOXP3,GATA3,IFNG,IL10,IL4,IL6,STAT6,TBX21
IL18		Activated	2.601	1.69E-13	CCL4,IFNG,IL10,IL1B,IL4,IL6,TNF
SASH1		Activated	2.449	4.26E-10	CCL4,CXCL10,IL12B,IL6,STAT1,TNF
ICOS		Activated	2.62	9.33E-16	GATA3,IFNG,IL10,IL4,IL6,TBX21,TNF
TLR9		Activated	2.803	3.8E-20	CCL4,CCL5,CXCL10,IFNG,IL10,IL12B,IL4,IL6,STAT1,TBX21,TNF
MAP2K3		Activated	2.191	1.73E-15	CXCL10,IFNG,IL10,IL12B,IL4,STAT1
KITLG		Activated	2.433	5.94E-12	CCL4,IFNG,IL1B,IL4,IL6,TNF
NFKB1		Activated	2.158	9.84E-18	ARG1,CCL17,IFNG,IL10,IL12B,IL1B,IL6,STAT1,TNF
IL15		Activated	2.931	1.24E-13	CCL17,CCL4,FOXP3,IFNG,IL10,IL12B,IL6,TBX21,TNF

IFNG	17.423	Activated	2.366	4.11E-24	ARG1,CCL4,CCL5,CCR5,CXCL10,FOXP3,IFNG,IL10,IL12B,IL1B,IL21,IL4,IL6,STAT1,TBX21,TNF
PRKD1		Activated	2.223	1.11E-20	CCL4,CCL5,CXCL10,IL10,IL12B,IL1B,IL6,TNF
TNFRSF1A		Activated	2.449	1.44E-13	GATA3,IFNG,IL1B,IL4,IL6,TBX21
FCER1G		Activated	2.169	1.58E-12	IL10,IL12B,IL1B,IL6,TNF
IL1B	5.278	Activated	2.198	1.83E-18	CCL4,CCL5,IFNG,IL10,IL12B,IL1B,IL6,TBX21,TNF
Klrk1		Activated	2.009	8.55E-17	CCL4,CCL5,CCR5,IFNG,IL10,STAT1,TBX21,TNF
PF4		Activated	2.398	2.76E-13	CCL22,CCL4,IFNG,IL1B,IL6,TNF
EBI3		Activated	2.207	1.58E-12	IFNG,IL10,IL21,IL4,TBX21
CD28		Activated	3.37	4.66E-27	CCL4,CCR4,CCR5,CXCR3,FOXP3,GATA3,IFNG,IL10,IL1B,IL21,IL4,IL6,STAT6,TBX21,TNF
TLR3		Activated	2.026	1.16E-15	CCL5,CXCL10,IFNG,IL10,IL12B,IL1B,IL6,STAT1,TNF
**LmCen$^{-/-}$**					
CARD9		Activated	2.204	1.1E-12	IL10,IL12B,IL1B,IL6,TNF
GPR84		Activated	2	2.51E-10	IL12B,IL1B,IL6,TNF
RNASE2		Activated	2.951	7.08E-21	CCL22,CCL4,CCL5,CXCL10,CXCL9,IL10,IL12B,IL6,TNF
CLEC6A		Activated	2.216	4.4E-15	IL10,IL12B,IL1B,IL6,TNF
DOCK8		Activated	2.449	4.36E-10	CCL4,CXCL10,IL12B,IL6,STAT1,TNF
IL33		Activated	2.776	2.14E-16	ARG1,CCL17,IFNG,IL10,IL1B,IL4,IL6,TNF
MAVS		Activated	2.592	1.43E-14	CCL5,CXCL10,IFNG,IL1B,IL6,STAT1,TNF
STING1		Activated	2.216	8.62E-10	CCL5,CXCL10,IL1B,IL6,TNF
CLEC7A		Activated	2.413	8.66E-15	CCL4,IL10,IL12B,IL1B,IL6,TNF
TLR7		Activated	3.018	2.92E-18	CCL4,CXCL10,CXCL9,IFNG,IL10,IL12B,IL1B,IL6,STAT1,TNF
STAT1	6.879	Activated	2.358	8.37E-26	CCL5,CXCL10,CXCL9,CXCR3,FOXP3,GATA3,IFNG,IL10,IL1B,IL21,IL6,TBX21,TNF
REL		Activated	2.209	3.17E-13	CCL4,CXCL10,FOXP3,IFNG,IL12B,IL1B,IL21,IL4,TNF
ITGA5		Activated	2	5.4E-11	IL12B,IL1B,IL6,TNF
STAT4		Activated	2.557	6.17E-13	CCR5,IFNG,IL10,IL6,STAT1,TBX21,TNF
TLR4		Activated	3.054	8.35E-16	CCL4,CCL5,CXCL10,IFNG,IL10,IL12B,IL1B,IL4,IL6,STAT1,TNF
ITGB1		Activated	2	7.53E-10	IL12B,IL1B,IL6,TNF
AKT1		Activated	2.449	6.22E-14	CCL5,CXCL10,FOXP3,IL10,IL6,STAT6
TLR2		Activated	3.071	9.59E-25	ARG1,CCL4,CCL5,CCR5,FOXP3,GATA3,IFNG,IL10,IL12B,IL1B,IL4,IL6,TNF
CD14		Activated	2.57	6.07E-18	CCL4,CCL5,CXCL10,IFNG,IL10,IL1B,IL6,TNF
IRAK4		Activated	2.024	6.36E-16	IFNG,IL10,IL12B,IL1B,IL4,IL6,TNF
STAT6	6.605	Activated	2.204	2.84E-16	ARG1,CCL17,CCR5,GATA3,IFNG,IL10,IL12B,IL4,IL6,STAT6,TBX21,TNF
SAMSN1		Activated	2.449	1.66E-09	CCL4,CXCL10,IL12B,IL6,STAT1,TNF
CDKN2A		Activated	2.044	1.61E-17	CCL22,CCL4,CCL5,CCR4,CXCL10,IFNG,IL1B,IL6,TNF
ICOSLG/LOC1027239		Activated	2.194	1.1E-12	FOXP3,IFNG,IL10,IL4,IL6
CSF2		Activated	2.567	2.35E-14	ARG1,CCL17,CXCL10,IL10,IL12B,IL1B,IL4,IL6,TNF
CD40LG		Activated	2.703	5.29E-16	CCL4,CXCL10,IFNG,IL10,IL12B,IL1B,IL6,TNF
NOD2		Activated	2.202	1.89E-11	CXCL10,IL10,IL12B,IL1B,IL6,TNF
S100A8		Activated	2.196	2.69E-11	CXCL10,CXCL9,IL1B,IL6,TNF
MYD88		Activated	3.083	1.98E-21	ARG1,CCL17,CCL4,CCL5,CXCL10,IFNG,IL10,IL12B,IL1B,IL4,IL6,TBX21,TNF
SYK		Activated	2.401	7.46E-10	CCL4,CXCL10,GATA3,IL1B,IL21,TNF
PRKCQ		Activated	2.603	1.8E-18	FOXP3,GATA3,IFNG,IL10,IL4,IL6,TBX21,TNF

SELPLG		Activated	2	1.43E-07	CCL4,IL10,IL1B,STAT6
TICAM1		Activated	2.381	1.66E-14	CCL4,CCL5,CXCL10,IL10,IL12B,IL1B,IL4,IL6,TNF
MAPKAPK2		Activated	2.156	1.61E-09	ARG1,CXCL10,IL10,IL6,TNF
FCGR2A		Activated	2.605	3.58E-15	CCL22,CXCL10,IFNG,IL10,IL1B,IL6,TNF
C3		Activated	2	4.52E-10	CCL4,IFNG,IL10,TNF
RNASE1		Activated	2.8	1.15E-18	CCL22,CCL4,CCL5,CXCL10,CXCL9,IL12B,IL6,TNF
GATA3	1.555	Activated	2.205	1.46E-17	CCL4,CCR5,FOXP3,GATA3,IFNG,IL10,IL4,IL6,STAT6,TBX21
CCL5	5.277	Activated	2.2	6.48E-09	CCL4,CCL5,IL1B,IL6,TNF
IL18		Activated	2.601	2.43E-13	CCL4,IFNG,IL10,IL1B,IL4,IL6,TNF
PTGS2		Activated	2.233	8.72E-12	CCL5,CXCL10,IL1B,IL6,TNF
TRIM24		Activated	2.219	2.02E-12	CCL4,CCL5,IL10,IL21,IL4
SASH1		Activated	2.449	5.74E-10	CCL4,CXCL10,IL12B,IL6,STAT1,TNF
ICOS		Activated	2.62	1.34E-15	GATA3,IFNG,IL10,IL4,IL6,TBX21,TNF
TLR9		Activated	3.342	3.42E-22	CCL4,CCL5,CXCL10,CXCL9,IFNG,IL10,IL12B,IL4,IL6,STAT1,TBX21,TNF
IRF3		Activated	2.2	3.13E-09	CCL5,CXCL10,IL10,IL4,STAT1,TNF
MAP2K3		Activated	2.401	1.82E-18	CXCL10,CXCL9,IFNG,IL10,IL12B,IL4,STAT1
KITLG		Activated	2.433	8.02E-12	CCL4,IFNG,IL1B,IL4,IL6,TNF
TLR6		Activated	2.588	9.46E-18	CCL5,IFNG,IL12B,IL1B,IL4,IL6,TNF
NFKB1		Activated	2.158	1.61E-17	ARG1,CCL17,IFNG,IL10,IL12B,IL1B,IL6,STAT1,TNF
IL15		Activated	2.931	2.02E-13	CCL17,CCL4,FOXP3,IFNG,IL10,IL12B,IL6,TBX21,TNF
IFNG	45.44	Activated	3.185	9.29E-26	ARG1,CCL4,CCL5,CCR5,CXCL10,CXCL9,FOXP3,IFNG,IL10,IL12B,IL1B,IL21,IL4,IL6,STAT1,TBX21,TNF
PRKD1		Activated	2.804	1.7E-20	CCL4,CCL5,CXCL10,IL10,IL12B,IL1B,IL6,TNF
TNFRSF1A		Activated	2.449	1.94E-13	GATA3,IFNG,IL1B,IL4,IL6,TBX21
BCL10		Activated	2.195	1.1E-12	CCL4,CCL5,IFNG,IL6,TNF
BECN1		Activated	2.208	2.02E-12	CCL5,IL12B,IL1B,IL6,TNF
FCER1G		Activated	2.169	2.02E-12	IL10,IL12B,IL1B,IL6,TNF
IL1B	16.219	Activated	2.758	3.01E-18	CCL4,CCL5,IFNG,IL10,IL12B,IL1B,IL6,TBX21,TNF
Klrk1		Activated	2.783	1.31E-16	CCL4,CCL5,CCR5,IFNG,IL10,STAT1,TBX21,TNF
PF4		Activated	2.398	3.73E-13	CCL22,CCL4,IFNG,IL1B,IL6,TNF
EBI3		Activated	2.207	2.02E-12	IFNG,IL10,IL21,IL4,TBX21
CD28		Activated	3.37	1.33E-26	CCL4,CCR4,CCR5,CXCR3,FOXP3,GATA3,IFNG,IL10,IL1B,IL21,IL4,IL6,STAT6,TBX21,TNF
TLR3		Activated	2.755	1.9E-15	CCL5,CXCL10,IFNG,IL10,IL12B,IL1B,IL6,STAT1,TNF

Supplementary Table 3 : Quality attributes and acceptance criteria for lot to lot consistency of GLP-grade *LmCen*$^{-/-}$ parasites

Quality Attribute	Acceptance criteria
**Physical Attributes**<br>**Morphology**	Ovoid cell body with a highly motile long flagellum
**Identification**<br><br>**Absence of Centrin gene**	PCR test negative & NGS analysis confirmed deletion of Centrin1 gene from the genome
**Purity**<br>**Test of sterility**	As recommended by the USP (https://www.uspnf.com/sites/default/files/usp_pdf/EN/USPNF/revisions/gc_1_rb_notice.pdf)
**Stability**	One-month post-cryo at Liquid N2: 70-75% viability<br>12-months post-cryo in Liquid N2: 70-75% viability
**Potency**<br>**Macrophage Infection Assay**	<20-30% parasite survival after 120 hours of infection in THP1 cell line
**Immune response**	TNF-a (1500-2000pg/mL)
**% of metacyclic promastigotes**	30-50%
**Content**<br>**Parasite content**	10^7 parasites/mL in 1.8mL per vial

Supplementary Table 4: Composition of cultivation medium of GLP-grade *LmCen*$^{-/-}$ parasites in Bioreactor

S. N.	Medium component	Stock concentration	Quantity/L
1	M 199	1 X	858 ml
2	PenStrep	100 X	10 ml
3	HEPES	1 M	25 ml
4	Hemin	5 g/L	2 ml
5	Adenosine	25 mM	4 ml
6	FBS (10% v/v)	-	100 ml
7	Folic acid	10 g/L	1 ml
8	Biotin	200mg/L	5 ml

Supplementary Table 5: Composition of Cryo-medium for GLP-grade *LmCen*$^{-/-}$ parasites stored in liquid nitrogen

S. N.	Medium component	Stock concentration	Quantity/L
1	M 199	1 X	858 ml
2	PenStrep	100 X	10 ml
3	HEPES	1 M	25 ml
4	Hemin	5 g/L	2 ml
5	Adenosine	25 mM	4 ml
6	FBS (10% v/v)	-	100 ml
7	Folic acid	10 g/L	1 ml
8	Biotin	200mg/L	5 ml
9	Glycerol (v/v)	-	10%

Supplementary Table 6: Primers and probe sequences used for the immunological analysis in hamsters

Hamster target gene	Primers and probes sequences	
**IL-12p40**	Forward	5'-AATGCGAGGCAGCAAATTACTC-3'
	Reverse	5'-CTGCTCTTGACGTTGAACTTCAAG-3'
	Probe	5'-(6FAM)-CCTGCTGGTGGCTGACTGCAATCA-(TAMRA)-3'
**IFN-γ**	Forward	5'-TGTTGCTCTGCCTCACTCAGG-3'
	Reverse	5'-AAGACGAGGTCCCCTCCATTC-3'
	Probe	5'-(6FAM) TGGCTGCTACTGCCAGGGCACACTC-(TAMRA)-3'
**TNF-α**	Forward	5'-TGAGCCATCGTGCCAATG-3'
	Reverse	5'-AGCCCGTCTGCTGGTATCAC-3'
	Probe	5'-(6FAM)-CGGCATGTCTCTCAAAGACAACCAG-(TAMRA)-3'
**IL-4**	Forward	5'-ACAGAAAAAGGGACACCATGCA-3'
	Reverse	5'-GAAGCCCTGCAGATGAGGTCT-3'
	Probe	5'-(6FAM) AGACGCCCTTTCAGCAAGGAAGAACTCC-(TAMRA)-3'
**IL-10**	Forward	5'-GGTTGCCAAACCTTATCAGAAATG-3'
	Reverse	5'-TTCACCTGTTCCACAGCCTTG-3'
	Probe	5'-(6FAM) TGCAGCGCTGTCATCGATTTCTCCC-(TAMRA)-3'
**IL-21**	Forward	5'-GGACAGTGGCCCATAAAACAAG-3'
	Reverse	5'-TTCAACACTGTCTATAAGATGACGAAGTC-3'
	Probe	5'-(6FAM)-CAAGGGCCAGATCGCCTCCTGATT-(TAMRA)-3'
**CCL17**	Forward	5'-GTGCTGCCTGGAGATCTTCA-3'
	Reverse	5'-TGGCATCCCTGGGACACT3'
	Probe	5'-(6FAM)-CCATTCCCATCAGGAAGCTGGTGATG-(TAMRA)-3'
**CCL22**	Forward	5'-TGGTGCCAACGTGGAAGAC-3'
	Reverse	5'-GAAGAACTCCTTCACTACGCGC-3'
	Probe	5'-(6FAM)-CTGCTGCCAGGACTACATCCGTCAGC-(TAMRA)-3'
**γ-actin**	Forward	5'-ACAGAGAGAAGATGACGCAGATAATG-3'
	Reverse	5'-GCCTGAATGGCCACGTACA-3'
	Probe	5'-VIC -TTGAAACCTTCAACACCCCAGCC-(TAMRA)-3'

Supplementary Table 6 : Primers and probe sequences (continues)

Hamster target gene	Primers and probes sequences	
IL-1β	Forward	5' –GGCTGATGCTCCCATTCG– 3'
	Reverse	5' –CACGAGGCATTTCTGTTGTTCA– 3'
IL-6	Forward	5' –CCTGAAAGCACTTGAAGAATTCC– 3'
	Reverse	5' –GGTATGCTAAGGCACAGCACACT– 3'
T-bet	Forward	5' –ACAAGGGGGCTTCCAACAAT– 3'
	Reverse	5' –CAGCTGAGTGATCTCGGCAT– 3'
GATA3	Forward	5' –GAAGGCAGGGAGTGTGTGAA – 3'
	Reverse	5' –GTCTGACAGTTCGCACAGGA– 3'
CXCR3	Forward	5' –CAAGTGCCAAAGCAGAGAAGC– 3'
	Reverse	5' –CAAAGTCCGAGGCATCTAGCA– 3'
CXCL9	Forward	5'-TGGGTATCATCCTCCTGGAC-3'
	Reverse	5'-AATGAGGACCTGGAGCAAAC-3'
CXCL10	Forward	5'-TGGAAATTATTCCTGCAAGTCA-3'
	Reverse	5'-GTGATCGGCTTCTCTCTGGT-3'
CCR4	Forward	5' –GCTTGGTCACGTGGTCAGTG– 3'
	Reverse	5' –GTGGTTGCGCTCCGTGTAG– 3'
CCR5	Forward	5' –TGTGACATCCGTTCCCCCT– 3'
	Reverse	5' –GGCAGGGTGCTGACATACTA– 3'
CCL4	Forward	5' –TCTCTCTCCTCCTGTTCGTGG– 3'
	Reverse	5' –TTTGCTTGCCTTTTCTGGTCA– 3'
CCL5	Forward	5' –CTACGCTCCTTCATCTGCCTC– 3'
	Reverse	5' –CCTTCGGGTGACAAAAACGAC– 3'
STAT1	Forward	5' –GCCAACGATGATTCCTTTGC– 3'
	Reverse	5' –GCTATATTGGTCATCCAGCTGAGA– 3'
STAT6	Forward	5' –GAAGCACCACTTTGCAACACA– 3'
	Reverse	5' –GGCAGGTGACGGAACTCTTCT– 3'
FOXP3	Forward	5' –AGGTCTTCGAGGAGCCAGAA– 3'
	Reverse	5' –GCCTTGCCCTTCTCATCCA– 3'
Arginase-1	Forward	5'-ACCTATGTGTCATTTGGGTGGA-3'
	Reverse	5'-GCAGATATGCAGGGAGTCACC-3'
18s	Forward	5'-ACCGCAGCTAGGAATAATGGA-3'
	Reverse	5'-GCCTCAGTTCCGAAAACCA-3'
