# Supplementary material for: Preclinical validation of a live attenuated dermotropic Leishmania vaccine against vector transmitted fatal visceral leishmaniasis
Source: Commun Biol. 2021 Jul 30;4:929. doi: 10.1038/s42003-021-02446-x (PMC8324786; doi:10.1038/s42003-021-02446-x)
Supplement: Supplementary file 3 — Reporting Summary [file 42003_2021_2446_MOESM3_ESM.pdf]

nature research

Corresponding author(s): Hira L. Nakhasi

Last updated by author(s): Jun 16, 2021

Reporting Summary

Nature Research wishes to improve the reproducibility of the work that we publish. This form provides structure for consistency and transparency in reporting. For further information on Nature Research policies, see our Editorial Policies and the Editorial Policy Checklist.

Statistics

For all statistical analyses, confirm that the following items are present in the figure legend, table legend, main text, or Methods section.

n/a	Confirmed	
☐	☒	The exact sample size (*n*) for each experimental group/condition, given as a discrete number and unit of measurement
☐	☒	A statement on whether measurements were taken from distinct samples or whether the same sample was measured repeatedly
☐	☒	The statistical test(s) used AND whether they are one- or two-sided<br>*Only common tests should be described solely by name; describe more complex techniques in the Methods section.*
☒	☐	A description of all covariates tested
☒	☐	A description of any assumptions or corrections, such as tests of normality and adjustment for multiple comparisons
☐	☒	A full description of the statistical parameters including central tendency (e.g. means) or other basic estimates (e.g. regression coefficient) AND variation (e.g. standard deviation) or associated estimates of uncertainty (e.g. confidence intervals)
☒	☐	For null hypothesis testing, the test statistic (e.g. *F*, *t*, *r*) with confidence intervals, effect sizes, degrees of freedom and *P* value noted<br>*Give P values as exact values whenever suitable.*
☒	☐	For Bayesian analysis, information on the choice of priors and Markov chain Monte Carlo settings
☒	☐	For hierarchical and complex designs, identification of the appropriate level for tests and full reporting of outcomes
☒	☐	Estimates of effect sizes (e.g. Cohen's *d*, Pearson's *r*), indicating how they were calculated

Our web collection on statistics for biologists contains articles on many of the points above.

Software and code

Policy information about availability of computer code

Data collection	*Provide a description of all commercial, open source and custom code used to collect the data in this study, specifying the version used OR state that no software was used.*
Data analysis	GraphPad Prism version 7.0 was used to generate graphs. Bio-Rad CFX Manager 3.1 for RT-PCR data analysis, Metaboanalyst 5.0 used for statistical analysis. Ingenuity Pathway Analysis was used for the analysis of immune marker data.

For manuscripts utilizing custom algorithms or software that are central to the research but not yet described in published literature, software must be made available to editors and reviewers. We strongly encourage code deposition in a community repository (e.g. GitHub). See the Nature Research guidelines for submitting code & software for further information.

Data

Policy information about availability of data

All manuscripts must include a data availability statement. This statement should provide the following information, where applicable:

- Accession codes, unique identifiers, or web links for publicly available datasets
- A list of figures that have associated raw data
- A description of any restrictions on data availability

The data that support the findings of this study are available from the corresponding author upon reasonable request.

Field-specific reporting

Please select the one below that is the best fit for your research. If you are not sure, read the appropriate sections before making your selection.

[x] Life sciences [ ] Behavioural & social sciences [ ] Ecological, evolutionary & environmental sciences

For a reference copy of the document with all sections, see nature.com/documents/nr-reporting-summary-flat.pdf

Life sciences study design

All studies must disclose on these points even when the disclosure is negative.

Sample size	In all experiments we have used at least 5 animals per group to perform Mann-Whitney two-tailed test analysis; no sample size calculation was necessary or performed since this study is of pre-clinical development stage.
Data exclusions	There was no data exclusions in this manuscript.
Replication	Where possible, all the experiments were replicated 2 times using different animal models, and methods of challenge and done one time using PBMCs of seven different healthy donors from non-endemic region were obtained from ATCC, USA (PCS-800-011™). We confirm that the attempts to replicate the findings are successful.
Randomization	In all experiments animals were randomly allocated between experimental groups
Blinding	Since this study is of pre-clinical development stage, blinding was not necessary or used in this study.

Reporting for specific materials, systems and methods

We require information from authors about some types of materials, experimental systems and methods used in many studies. Here, indicate whether each material, system or method listed is relevant to your study. If you are not sure if a list item applies to your research, read the appropriate section before selecting a response.

Materials & experimental systems

n/a	Involved in the study	
[ ]	[x]	Antibodies
[ ]	[x]	Eukaryotic cell lines
[x]	[ ]	Palaeontology and archaeology
[ ]	[x]	Animals and other organisms
[ ]	[x]	Human research participants
[x]	[ ]	Clinical data
[x]	[ ]	Dual use research of concern

Methods

n/a	Involved in the study	
[x]	[ ]	ChIP-seq
[x]	[ ]	Flow cytometry
[x]	[ ]	MRI-based neuroimaging

Antibodies

Antibodies used	Rabbit anti-hamster IgG1-HRP, Rabbit anti-hamster IgG2a-HRP; Southern Biotech, Birmingham, AL
Validation	We followed manufacturer's instruction

Eukaryotic cell lines

Policy information about cell lines

Cell line source(s)	L. major Friedlin (FV9), L. donovani (LdWT) (MHOM/SD/62/1S)
Authentication	L. major Friedlin (FV9) and L. donovani (LdWT) (MHOM/SD/62/1S) confirmed to be L. major and L. donovani through genome sequening
Mycoplasma contamination	Not tested
Commonly misidentified lines (See ICLAC register)	No misidentified cell lines used

Animals and other organisms

Policy information about studies involving animals; ARRIVE guidelines recommended for reporting animal research

Laboratory animals	Six to eight-week-old female outbred Syrian golden hamsters (Mesocricetus auratus), Female Lutzomyia longipalpis (Jacobina strain) are reared at the Laboratory of Malaria and Vector Research, NIAID/NIH.
Wild animals	No wild animals were used in this study
Field-collected samples	No field collected samples were used in this study
Ethics oversight	The animal protocol for this study has been approved by the Institutional Animal Care and Use Committee at the Center for Biologics Evaluation and Research, US FDA (ASP 1999#23). The animal protocol is in full accordance with "The guide for the care and use of animals as descried in the US Public Health Service policy on Humane Care and Use of Laboratory Animals 2015".<br>Animal experimental procedures performed at the National Institute of Allergy and Infectious Diseases (NIAID) were reviewed by the NIAID Animal Care and Use Committee under animal protocol LMVR4E. The NIAID DIR Animal Care and Use Program complies with the Guide for the Care and Use of Laboratory Animals and with the NIH Office of Animal Care and Use and Animal Research Advisory Committee guidelines. The housing condition of animals were followed standard guidelines by NIH guidelines for the humane care and use of animals.

Note that full information on the approval of the study protocol must also be provided in the manuscript.

Human research participants

Policy information about studies involving human research participants

Population characteristics	PBMCs of seven different healthy donors from non-endemic region were collected from ATCC, USA (PCS-800-011™). The anonymous donors that meet the blood donation criteria and CMV negative samples were used. We had no role in defining the donor inclusion criteria.
Recruitment	Since PBMCs of seven different healthy donors from non-endemic region were collected from ATCC, USA, we did not have any role in recruitment.
Ethics oversight	An institutional committee at the US FDA (Research Involving Human Subjects Committee, RIHSC) reviewed the protocol and approved the study prior to the start of the experiments. The study was reviewed biannually by the RIHSC.

Note that full information on the approval of the study protocol must also be provided in the manuscript.
